# Mapping state-sponsored information operations with multi-view modularity clustering

**DOI:** 10.1140/epjds/s13688-022-00338-6

**Published:** 2022-04-18

**Authors:** Joshua Uyheng, Iain J. Cruickshank, Kathleen M. Carley

**Affiliations:** CASOS Center, Institute for Software Research, Carnegie Mellon University, 5000 Forbes Ave, 15213 Pittsburgh, PA USA

**Keywords:** COVID-19 pandemic, Information operations, Multi-view modularity clustering, State-sponsored disinformation, Social cyber-security, Unsupervised machine learning

## Abstract

This paper presents a new computational framework for mapping state-sponsored information operations into distinct strategic units. Utilizing a novel method called multi-view modularity clustering (MVMC), we identify groups of accounts engaged in distinct narrative and network information maneuvers. We then present an analytical pipeline to holistically determine their coordinated and complementary roles within the broader digital campaign. Applying our proposed methodology to disclosed Chinese state-sponsored accounts on Twitter, we discover an overarching operation to protect and manage Chinese international reputation by attacking individual adversaries (Guo Wengui) and collective threats (Hong Kong protestors), while also projecting national strength during global crisis (the COVID-19 pandemic). Psycholinguistic tools quantify variation in narrative maneuvers employing hateful and negative language against critics in contrast to communitarian and positive language to bolster national solidarity. Network analytics further distinguish how groups of accounts used network maneuvers to act as balanced operators, organized masqueraders, and egalitarian echo-chambers. Collectively, this work breaks methodological ground on the interdisciplinary application of unsupervised and multi-view methods for characterizing not just digital campaigns in particular, but also coordinated activity more generally. Moreover, our findings contribute substantive empirical insights around how state-sponsored information operations combine narrative and network maneuvers to achieve interlocking strategic objectives. This bears both theoretical and policy implications for platform regulation and understanding the evolving geopolitical significance of cyberspace.

## Introduction

Coordinated actors use information maneuvers to shift public narratives and alter the information flow between individuals and groups [1–4]. The impacts of such campaigns reverberate not only through cyberspace, but also in high-stakes, real-world settings [5–7]. Using previously labeled data, machine learning tools abound for automatically identifying potential misinformation, rumors, bot accounts, hate speech, and other elements which may attend attempts to manipulate public discourse [8–11]. Network science likewise provides systematic frameworks for representing complex patterns of online communication [7, 12]. This facilitates the principled identification of actors holding various types of influence over the conversation, acting as hubs for information, bridges between groups, or trying to break groups apart [13–15].

However, many frameworks developed focus on specific components of information operations in isolation [1, 2, 16]. Compelling inquiry pertains to how a larger information operations group may use all of these different approaches as part of a single campaign. As information operations unfold in real time, they achieve their objectives in multiple, dynamic ways. It may not be known in advance which analyses are most relevant to which facets of the campaign. Hence, while existing models – especially those founded on supervised machine learning algorithms – may generate rich and accurate predictions on precise dimensions of an information operation, they are not designed to account for the multifaceted and novel nature of influence campaigns as a whole [12, 17, 18]. Likewise, existing unsupervised approaches may tend toward distinguishing *between* clusters of malicious versus benign actors. In contrast, little work has been done in mapping the internal structure *within* a collection of adversarial accounts [9, 19, 20].

This paper addresses these gaps in the literature by presenting *multi-view modularity clustering* as an unsupervised, data-driven methodology for characterizing information operations. Here, we detect coordinated groups of accounts by simultaneously considering their attributes, their behaviors, as well as their interactions [21]. Common approaches to clustering may tend to present an exclusive focus on just networks of interactions between accounts or just the text being emitted by accounts [19, 20]. As such, if a given account does not engage in a certain type of interaction or post any text, they cannot be effectively clustered by that one source of information. So, using more views of the information operation actors can provide richer insight to not only provide better clusters, but also address issues like partially complete views of the data [16]. In addition, we also present principled post-hoc measures for uncovering key characteristics of the derived clusters and their interrelationships. As our findings demonstrate, these methods are vital to taking advantage of the generated cluster assignments by probing their structural and functional relevance to the broader setting of information operations.

Utilizing a Twitter dataset of disclosed state-sponsored actors, we show how data-driven characterizations of information operation clusters can shed light on how digital campaigns organize tasks and undertake information operations through narrative and network maneuvers. This facilitates empirical mapping of varied and integrated tactics in information maneuvers, without relying on extensive and expensive labeled data [22]. This information is vital in helping platforms and governments to better identify and respond to digital campaigns as they emerge against the intersecting and volatile contexts of cyberspace and geopolitics [1, 5, 6, 23].

In sum, this work therefore aims to answer the following questions:

1. How can we identify, in an unsupervised manner, meaningful clusters of information operation actors?
2. How can we characterize these clusters to identify distinct narrative and network maneuvers within state-sponsored information operations?

## Related work

### The supervised paradigm in social cyber-security

The empirical literature on digital campaigns – collectively subsumed within the domain of social cyber-security – has flourished within the broader, interdisciplinary sphere of computational social science [2, 6]. Over the past decade, large-scale investigations into the prevalence and activity of coordinated agents online has particularly been facilitated by the surge in *supervised methods* for computational modelling, which identify new malevolent actors based on cumulative evidence from prior experience [24, 25]. Within this methodological framework, researchers develop and train predictive models based on labeled instances of so-called bots, trolls, or cyborgs, and apply these models to new empirical cases [8, 10, 11]. The characteristics of these identified accounts may then be systematically evaluated across relevant case studies of interest to inform counter-strategies for enhanced societal resilience to information operations writ large [12, 26–28].

This supervised paradigm of scholarship has uncovered valuable insights about the impacts of information operations, spanning a range of domains like politics [5, 29], finance [30, 31], and public health [14, 32]; as well as in diverse national and international contexts around the world [12, 33–36]. Across this vast literature, important findings include the quantification of links between the activity of social bots and the spread of low-credibility information or fake news [37]; how automated accounts increase human exposure to more inflammatory content [4]; how bots can spread hate most effectively in groups that are denser and more isolated from mainstream dialogue [7]; and how they may even attenuate the influence of more traditional opinion leaders in online conversations [15].

### Identifying information maneuvers in information operations

Notwithstanding the vast diversity of digital tactics and contexts worldwide, a synthesis of the literature reveals tendencies toward similar strategies that disinformation agents may adopt in achieving operational goals. At this juncture, we distinguish between (a) *information maneuvers*, which we use to refer to concrete patterns of empirically observable activity to influence narratives or networks in an online conversation; and (b) *information operations*, which comprise the wider programmatic collection of actors and the maneuvers they implement in pursuit of a broader, more abstract, overarching goal. Our core proposition is that within a given information operation, we may valuably discern multiple information maneuvers which act in a complementary fashion to achieve an overarching tactical objective in cyberspace.

Beskow and Carley [13] summarizes these information maneuvers in seminal work that distinguishes between *narrative maneuvers*, which generally comprise message-level tactics by disinformation agents that may be measured through their use of language and other textual artifacts; and *network maneuvers*, which refer to strategies of interaction between actors that contribute to shifts (or stability) in the flows of information and influence throughout the online conversation. This conceptual framework synthesizes and aligns with a diverse collection of research strands which examine the framing strategies of information operations and their concerted control of information flow to shape who hears what message and from whom [5, 14, 36, 38].

Narrative maneuvers may include constructive procedures such as *explaining* or *enhancing* messaging around a topic, or destructive procedures such as *dismaying* opposed groups or *distracting* the public conversation from salient aspects of a given topic. Network maneuvers are likewise divided into constructive actions that *build* a group or *boost* an influencer, and destructive tactics aiming to *nuke* groups to diminish their size and influence or *neglect* others from the conversation. While qualitative analysis of such maneuvers has resulted in rich insights into the diversity of information operations [39, 40], they have primarily been driven by supervised methods that face important limitations regarding the evolution and innovation of new influence campaigns, as we review in the next section.

### Evolving information operations and the need for unsupervised models

Despite the utility of the foregoing supervised paradigm, critiques have also arisen following real-world shifts in the increasingly sophisticated conduct of information operations [17, 41]. As supervised technologies grew more adept at detecting previously encountered variants of disinformation agents, the agents deployed in new influence campaigns began to evolve and deviate from known behaviors. This introduced a challenging chicken-and-egg problem for the computational social science of digital campaigns. Empirical insights based on the well-established supervised paradigm became difficult to apply to emergent cases which – by design – violated historically informed, cumulative scientific knowledge.

For instance, even within the same Reddit fora, researchers discovered that trolls tended to shift link-sharing behaviors to evade detection [42]. In the 2020 Canadian federal elections, bots used creative strategies of explicitly claiming *not* to be bots, thereby not only creating confusion about their own identities, but also shifting burdens of proving authenticity to human participants in the online conversation [43]. More general analysis has also found a persistent false positive problem in the application of bot detection algorithms, especially in contexts where disinformation agents came from distinct settings from the domain of an algorithm’s training datasets [18].

In this view, a parallel wave of research into digital campaigns has focused on unsupervised methods. This body of work has particularly emphasized clustering approaches that do not rely on labeled datasets of limited size and generalizability over time. Clustering approaches instead probe the actual features and behaviors of each new dataset they encounter. Then, based on patterns of shared and unshared properties among subsets of the dataset, they identify meaningful groups of data points. Past studies have looked into such methodologies by harnessing a diverse set of techniques, including warped correlation, network random walks, and high-dimensional document embeddings [9, 19, 20].

From this standpoint, social cyber-security researchers and practitioners have also approached the question of finding digital campaigns by looking at anomalous groups of accounts based on the derived clusters, rather than relying on individually classified individuals. From a practical perspective, this framework allows for more confident assessments robust to accumulated error rates from independently generated individual-based predictions. Moreover, group-level predictions capture the well-studied theoretical notion that digital campaigns operate in a coordinated fashion [2, 7], while also remaining less easily disrupted by innovative deviations from past behaviors [12, 17].

### Multi-view modularity clustering

In light of the benefits of clustering techniques, the present research now takes up this ongoing challenge of unsupervised characterization of information operations. More specifically, we argue that *a multi-view approach* is beneficial for account clustering in social cyber-security, for a combination of interlocking theoretical, methodological, and empirical reasons which we outline below [16, 21].

Multi-view clustering techniques are techniques designed to handle clustering of objects which can be described by more than one data source. Many different real-world, social phenomena give rise to ‘views’ of data which are often different types of data that can be used to describe the same set of actors. For example, social media accounts can post content, which could give rise to a text view. Accounts may also have interactions with each other, which can give rise to network views. Multi-view clustering aims to fuse the information from these different views of the data to produce one clustering of the objects that created the data [16, 44–46].

Of the many proposed approaches to multi-view clustering, those that belong to the family of techniques known as *intermediate integration* tend to be the most successful [16, 22]. Intermediate integration techniques generally rely on mapping all of the data to a compatible format without losing view-specific properties, and then collectively cluster all these views. These are contrasted against *early integration techniques*, where features are transformed for convenient clustering with a single technique, and may fall short of capturing nuances in distinct feature types; and *late integration techniques*, where integration is conducted after separately conducted clustering algorithms, and may ignore important correlations in group structure across feature types [21].

Empirically, the use of graphs as an intermediate data format has shown great success in multi-view clustering in a variety of domains [16, 46, 47]. There has been a surge of new techniques developed in multi-view clustering for handling genetic data [48, 49], image data [16, 50], and more recently human, social-based data [21]. Recent research with hashtags on Twitter during the COVID-19 pandemic has found multi-view clustering to be an effective means of characterizing topical discussion groups [22]. So, multi-view clustering can be used as a means of finding richer clusters from real-world data, than just clustering any particular view of the data by itself.

### Our contribution: a multi-view mapping of state-sponsored information operations

We therefore bring multi-view clustering techniques to bear on social cyber-security by positing that multi-view clustering techniques are methodologically desirable for integrating diverse data types which capture individual-level and group-level behavior. Theoretically, such diverse features are associated not just with social media in general, but also information maneuvers in particular. Furthermore, from an empirical standpoint, we demonstrate that multi-view clustering is helpful not just for distinguishing inorganic accounts from human accounts, as has been the major focus in existing literature as reviewed above. Rather, we show how multi-view clustering can be novelly used to detect and map out organized narrative and network maneuvers *within* a given set of coordinated disinformation actors [1, 2, 13].

In mobilizing this analytical shift, we therefore affirm growing efforts to harness unsupervised methods in the study of digital campaigns. But beyond this, we also refine ongoing preoccupations with groups of accounts rather than individual agents. In particular: (a) we push outward to *groups of groups* as our locus of analysis, that is, we frame state-sponsored information operations as supersets of meaningful clusters of accounts; and (b) we enhance our focus on coordination by using clustering not only to maximize differences between derived groups, but also to map out their interrelationships.

Taken together, these insights lie at the core of the present paper’s contributions, which we summarize as follows:

1. we enhance the theoretical utility of unsupervised modelling of information operations, using the recently developed multi-view modularity clustering (MVMC) technique;
2. we design and apply a data-driven methodological framework for deriving meaningful clusters of state sponsored accounts as well as post-hoc characterization procedures that link derived account groups to specific information maneuvers;
3. and finally, we present novel empirical findings around the organization of (Chinese) state-sponsored information operations.

## Data and methods

In this section, we present the details of *multi-view modularity clustering* alongside a novel framework for post-hoc characterization of the derived account clusters to map information maneuvers. We also introduce the data we used on Chinese state-sponsored actors to demonstrate the utility of our technique. Although, to prioritize a balance of depth and parsimony, this paper focuses on a single set of empirical results, we also emphasize how these techniques may be readily extended to any relevant set of coordinated agents featuring a wide range of individual-level and group-level features and interactions [21, 22].

### Data on state-sponsored actors

We apply our proposed framework to the Information Operations dataset disclosed by Twitter around known state-sponsored actors associated with China (June 2020).^1^ The data consisted of over 23,000 accounts, including their individual account features, the tweets they generated, and their patterns of interaction with each other and with other accounts.

**Figure 1:**
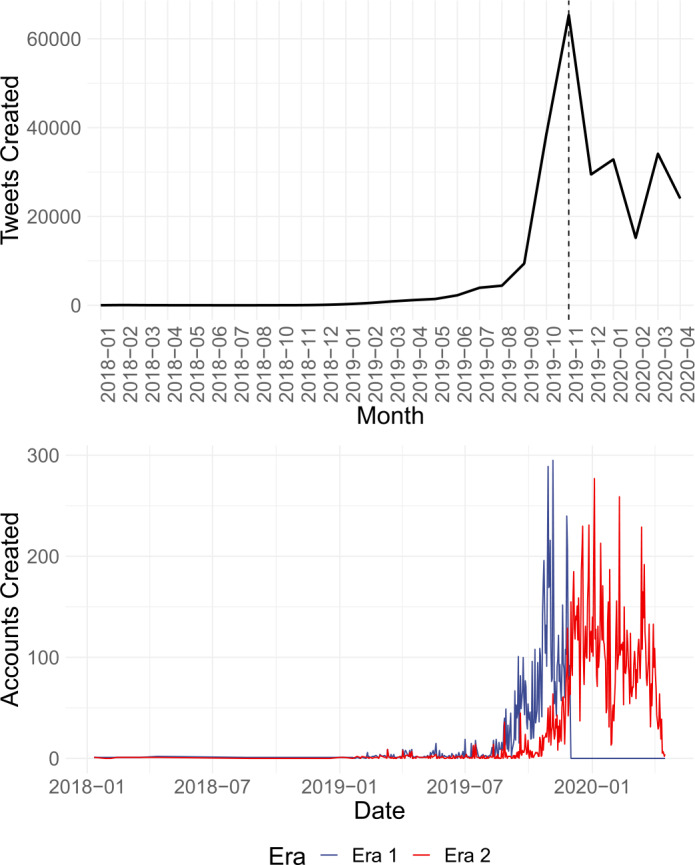
Tweet creation and account creation times of state-sponsored accounts. *Top:* A spike in tweet creation is observed in November 2019. *Bottom:* Accounts active before the spike (Era 1) are older than those active after it (Era 2)

Although Twitter released this corpus in a single round of disclosures, we observed a distinct spike in tweet production by these accounts around November 2019, as depicted in Fig. 1. For this reason, we decided to divide the dataset into two subsets: one for *before* (henceforth, Era 1), and one for *after* this uptick (henceforth, Era 2). This allowed us to explore questions not only of mapping the internal structure of a given collection of state-sponsored accounts; but also of discovering how their organization and information campaign efforts may have shifted with time. Empirical distributions of account creation times, also in Fig. 1, further showed that accounts which tweeted after the spike were largely created with extreme recency, thereby lending additional motivation to perform separate clusterings for the two eras. We note that this spike coincides with significant intensification of crackdowns on Hong Kong protests in November 2019.^2^

Descriptive statistics for the accounts analyzed are also provided in Table 1, showing that many tweets produced by these accounts were retweets rather than original messages, and they tended to follow more accounts rather than being followed themselves, and overwhelmingly tweeted more in Chinese rather than in English across time periods.

**Table 1:** Descriptive statistics for Chinese state-sponsored accounts. Statistics for Followers and Following are given as the mean and the range in brackets. Percentages are provided for retweets in each era, as well as percentage of tweets in Chinese/English. The remaining minority of tweets were in other languages or were not identified by Twitter

Era	Followers	Following	Retweets	Language (Chinese/English)
1	2.837 [0,806]	4.375 [0,1556]	68.12%	85.91%/6.19%
2	2.100 [0,806]	2.806 [0,1556]	68.51%	79.11%/11.98%

### General methodology: ORA

We used the ORA software to handle general network data management, network analysis techniques, and network data visualization.^3^ While we describe them in explicit mathematical terms below, processes including the MVMC algorithm are all available in the off-the-shelf software [51]. Note that other network analytic software are available to perform many of the general procedures described below; ORA, however, is specifically designed with multi-view modularity clustering and its subsequent procedures to characterize information maneuvers in mind. Hence, we characterize the succeeding methodological descriptions in terms of ORA for specificity and reproducibility.

### Constructing network views

Since the data presented has many different ways of analyzing the state-sponsored accounts’ behavior, we have elected to treat the analysis of patterns of users as a multi-view clustering problem. In general multi-view clustering seeks to produce one set of clustering labels for a set of objects, based upon multiple types or views of data about those objects [21].

To pre-process our dataset for multi-view modularity clustering, we enriched and organized the data according to several network views. A view refers to a set of behaviors or characteristics which organize the interrelationships between accounts within a network structure [16, 21]. For this study, we looked at three different views of characterizing agent behavior: the agent interactions with other agents, the text agents tweet, and the hashtags that the agents tweet. These are summarized in Table 2.

**Table 2:** Summary of network views used to process state-sponsored actors for multi-view modularity clustering

View	Node (From)	Node (To)	Edge Weights
Account Interaction	Twitter Account	Twitter Account	Sum of the number of shared accounts that those accounts mention, reply to, or retweet
Account Text	Twitter Account	Tweet Text	Counts of psycholinguistic features based on words used by accounts across all their tweets
Account Hashtags	Twitter Account	Hashtags	Counts of the hashtags that accounts use in all of their tweets

Account interaction networks refer to the patterns of communication between accounts that occur on Twitter. Here, we specifically consider three types of Twitter-based interaction: mentions, replies, and retweets. Each interaction type comprises a network view of the data. For instance, the mention network will consist of nodes representing all the accounts in the dataset. Edges represent mention relationships between accounts, with a given edge from account A to account B assigned a weight equal to the number of times account A mentions account B. Similar constructions are applied to replies and retweets.

Next, account text networks refer to psycholinguistic patterns in the messages sent by accounts [52]. Here, we use the Netmapper software^4^ to compute lexical counts of these psycholinguistic features with a dictionary approach for all tweets produced by the state-sponsored accounts [53]. Including theoretically formulated and multilingual measurements of pronoun usage, abusive words, emotional words, absolutist and exclusive words, identity words, and reading difficulty, these counts have been empirically shown to be predictive of bot-like language and hate speech across a range of information operation contexts [7, 35, 54, 55]. The account text network thus consists of a bipartite network of account nodes connected to nodes representing each psycholinguistic feature, with edge weights given by how much each feature is present (on average) in the account’s tweets. As before, other text analytic software and techniques may be available to achieve similar purposes; however, Netmapper specifically works with the multi-lingual functionality which suit our aims and the dataset of interest, so we specify our methodological description around Netmapper for reproducibility.

Finally, account hashtag networks refer to another bipartite network, between account nodes and nodes representing each unique hashtag used in the entire corpus. Here, the edges are weighted by the average usage counts of each hashtag; that is, the average number of times a given account uses a given hashtag.

Utilizing these text, hashtag, and interaction graphs, each of these views is then transformed into a new graph by a symmetric k-Nearest Neighbor Graph (k-NN). Here, the number of nearest neighbors is chosen to be $k=\sqrt{n}$, where *n* is the number of objects (i.e., accounts) being clustered, and cosine similarity was used to measure account similarity across views [56, 57]. To symmetrize the k-NN, the average strategy, $A'=\frac{1}{2} (A + A^{T})$, which is common in spectral clustering methods [58, 59], was used to produce the final view graph. Descriptive statistics and topologies for unimodal representations of each network view are summarized in Table 3.

**Table 3:** Descriptive statistics for unimodal representations of each network view across time periods analyzed

Era	View	Size	Density	Mean Link Weights
1	Account Interaction	8268	3.334 × 10^−4^	4.3406
	Account Text	8268	0.551	1.517
	Account Hashtags	8268	0.431	1.459
2	Account Interaction	16,661	1.292 × 10^−4^	3.756
	Account Text	16,661	0.448	1.599
	Account Hashtags	16,661	4.527 × 10^−4^	3.064

### Identifying account clusters with MVMC

**Figure 2:**
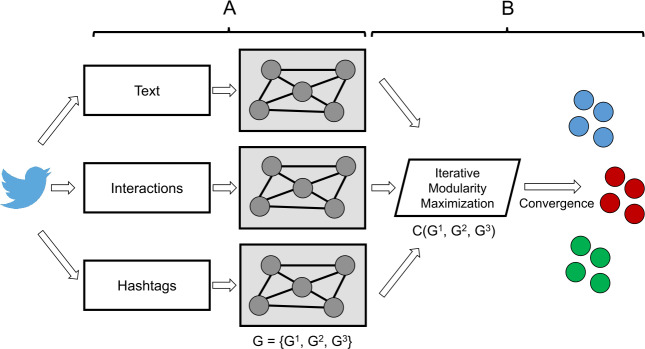
Graphical depiction of the MVMC technique used in this study. In the first step of the method, **A**, a graph representation is learned for every view of the data. In the second step, **B**, the view graphs are all collectively clustered to produce a single clustering across all of the views. Figure adapted from prior foundational work on MVMC [21, 22]

These k-NN view graphs serve as inputs to the multi-view clustering technique of Multi-view Modularity Clustering (MVMC).^5^ MVMC is a technique designed to work with multiple views, of any data type, of the same underlying social-based phenomena to produce a single set of clusters [21]. Figure 2 provides a graphical depiction of the overall process involved in this multi-view clustering technique.

The technique works in two main steps. First, a graph is learned for every view of the data, then an iterative procedure clusters all of the view graphs by optimizing a view-weighted, resolution-adjusted modularity function, given by Equation (1) below:

1 $$ Q = \sum_{v = 1}^{m} w^{v} \sum_{i,j \in E^{v}} \biggl[A_{ij}^{v} - \gamma ^{v} \frac{\deg (i)^{v} \times \deg (j)^{v}}{2\sum E^{v}} \biggr]\delta (C_{i},C_{j}), $$

where, for a particular network view *v*, $A^{v}$ represents the view’s adjacency matrix (which may be weighted), $\deg (i)^{v}$ denotes the degree of node *i*, *E* is the set of edges, and *δ* is the delta function for whether nodes *i* and *j* are assigned to the same cluster *C*.

Here, the modularity objective *Q* resembles the seminal definition of (multi-layer) network modularity [60], where we take a sum over different views (denoted by *v*) of a complex network. As explained in Cruickshank et al. [22], two key extensions are crucial for applying this technique to social-based data, especially information operations. First, the novel parameter $w^{v}$ represents a view-specific weight such that more important views that contribute to overarching group structure are assigned higher weights than others. Second, the parameter $\gamma ^{v}$ enables the algorithm to adjust to different resolution limits in clustering certain network views given their relative sizes and differences in topology. Values of $w^{v}$ and $\gamma ^{v}$ in this work are derived empirically in the iterative optimization process, following guidance from previous work [22].

This approach identifies structural and functional components of accounts based on both the text they emit and their patterns of interaction. Key benefits of MVMC lie not only in its unsupervised learning of the clusters themselves, but also of its adaptation of clustering heuristics to existing view-level network properties related to overarching latent community structure. This methodological design boosts the advantages of an MVMC approach in analyzing potentially innovative disinformation strategies. Further mathematical details about the multi-view objective function and its corresponding iterative learning algorithm are presented in prior work that introduces this technique [21, 22].

### Mapping information maneuvers

To further enhance the theoretical utility of MVMC in the social cyber-security space, we propose a framework for characterizing the coordinated behaviors associated with the identified account clusters. These steps represent a novel set of principled computational procedures for linking empirically derived groups of accounts with theoretically motivated features of information maneuvers.

**Figure 3:**
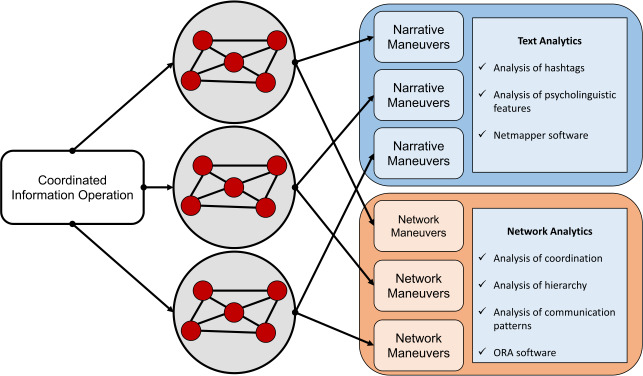
Conceptual framework of relationship between overarching information operations, specific narrative and network maneuvers enacted by account clusters, and the methods to characterize them

Figure 3 represents our underlying conceptualization of how information operations writ large serve as an organizing core to cluster-level narrative and network maneuvers [1, 2]. Here, for example, while we consider the Twitter dataset of Chinese state-sponsored accounts as participating in a common information operation, our goal now is to meaningfully characterize the MVMC-derived clusters to gain a better sense of how these accounts may be organized toward their strategic objectives.

The following methods are thus designed with this overarching conceptual goal in mind. Through text analytical methods, we aim to identify the information operation’s core messaging strategies; and through network analytics, we measure their levels of coordination, hierarchy, and communication patterns for shaping information flow. ORA also performs measurements automatically [51], following the taxonomy of maneuvers outlined in seminal work [8].

#### Visualizing overall cluster organization

To operationalize our “group of groups” understanding of information operations, we first visualize the derived clusters within a new network structure. Here, nodes of the cluster network will represent MVMC clusters. An edge between cluster *i* to cluster *j* will be weighted according to the number of interactions agents assigned to cluster *i* direct toward agents assigned to cluster *j*.

The utility of this procedure lies not only in its heuristic benefit, but also facilitates precise computations about the interrelationships of account clusters with each other. In particular, using standard measures of centrality – for which we simply use total degree and betweenness centrality – we can determine which aspects of the broader information operation serve as part of a core strategic effort or may play more of a supporting role in the network periphery.

#### Discovering narrative lines of effort

To qualify the narrative lines of effort associated with each MVMC cluster, we use straightforward computations of hashtag usage among accounts assigned to each cluster. Here, we posit that the most frequently used hashtag by accounts in a cluster signal key messages that the given cluster may seek to amplify or influence. While more advanced natural language processing techniques may be applied at this stage, we opt to use hashtags as the most general anchor for messaging analysis which cuts across languages and is broadly applicable to Twitter and similar platforms. Hashtags have likewise been used to similar effect in unpacking messaging aims of information operations in prior work [29, 43, 54].

For parsimony, we list the top 10 hashtags with the highest frequency of occurring among tweets generated by accounts in a given cluster. Further, noting that because all accounts may share common hashtags as part of a common information operation, we also highlight which among the top 10 hashtags offer distinct evidence regarding what differentiates clusters from each other. To facilitate this, we use a formulation of the classical tf-idf (term frequency-inverse document frequency) [61]. In particular, as expressed in Equation (2) below, we compute the tf-idf score $w_{i,j}$ of hashtag *i* used in a given cluster *j* as follows:

2 $$ w_{i,j} = \log (tf_{i,j} + 1) \times \log \frac{ \vert D \vert }{\sum_{j' \in D} \mathbb{I}(i \in d_{j'})}, $$

where $tf_{i,j}$ denotes the number of times hashtag *i* occurs is used by cluster *j*, *D* represents the set of all clusters, $d_{j'}$ represents the set of all hashtags used by arbitrary cluster $j'$, and $\mathbb{I}(\cdot )$ denotes the indicator function. By presenting the top 10 most used hashtags and then flagging which among them have the highest tf-idf scores, we balance objectives of discovering narrative lines of effort featuring both magnitude and distinctiveness within the broader information operation.

#### Mapping linguistic variation

While hashtags shed light on the substantive messaging topics of a given information operation, it is also valuable to examine the psycholinguistic features with which such messages are actually communicated. Here, we draw on the psycholinguistic literature that links the use of different aspects of language to cognitive and affective states, as well as strategies of persuasion, deception, and aggression [52, 54]. Here, we return to the Netmapper features as described earlier. In this context, we might wish to know whether some clusters speak about the same topic (e.g., “#HongKong”) in ways that are associated with positive or negative sentiment, using simpler or more complex language, or with the presence or absence of abusive language. This enhances our understanding of narrative maneuvers as it allows us to identify, in a theoretically motivated manner, whether accounts are seeking to shift emotions around a topic, amplify conflicts, simplify discussions, among other potential strategies.

To produce this characterization, we utilize principal component analysis (PCA) [62]. First, we construct a cluster by feature matrix, where each row corresponds to a given MVMC cluster, and each column is associated with a psycholinguistic feature of interest. Each entry $x_{i,j}$ in the matrix corresponds to the average score accounts of cluster *i* on psycholinguistic feature *j*. Then, through PCA, we produce a lower-dimensional representation of this matrix that captures the most meaningful sources of variation in the data, and allows us to parsimoniously map key associations between clusters and psycholinguistic features. Note that other methods may be applied to similar ends, such as t-distributed stochastic neighborhood embedding (t-SNE); however, we opted for PCA for parsimony and linearly interpretable dimension reduction.

#### Identifying account hierarchies

Now shifting to network maneuvers, one of the first aspects we focus on is the level of hierarchy within each cluster. To quantify this, we first use ORA to measure the total-degree centrality of every agent within its cluster, based on a sum of all Twitter mentions, replies, and retweets. This captures the extent to which each agent receives and generates messages to and from other agents in the cluster, and serves as a straightforward measurement of its role in group-level information flow.

We then compute the association between total-degree centrality and the number of followers each account has through regression analysis. In particular, for an account *i* in cluster *c*, we estimate the value of a Follower-Hierarchy Coefficient $\beta _{c} = \beta _{1} + \beta _{1,c}$, which we model using Equation (3) below:

3 $$ t_{i} = \beta _{0} + \beta _{1} f_{i} + \sum_{c'} \beta _{1,c'} \mathbb{I}\bigl(i \in c'\bigr) + \epsilon , $$

where $f_{i}$ refers to the number of followers account *i* possesses, $t_{i}$ is account *i*’s total-degree centrality, $\mathbb{I}(\cdot )$ is the indicator function, and *ϵ* refers to the error term of the regression equation. For ease of interpretation, we linearly scale the values of $\beta _{c}$ to the interval from 0 to 1, using Equation (4) to obtain normalized coefficients $\beta _{c}^{*}$:

4 $$ \beta _{c}^{*} = \frac{\beta _{c} - \min_{c'} \beta _{c'}}{\max_{c'} \beta _{c'} - \min_{c'} \beta _{c'}}. $$

We propose here that account clusters *c* featuring the highest values of $\beta _{c}^{*}$ are those where there are clear leader-follower structures. Lower values of $\beta _{c}^{*}$ indicate less of such a correspondence, and may signal more egalitarian tactics, or perhaps the lack of discernible vertical organization altogether. We use this measure as follower-following patterns offer important insight into the designated network role of each individual actor. However, Twitter disclosures do not provide the entire follower-following network of the state-sponsored accounts, so network computations cannot be conducted on the follower-following network directly. This straightforward use of regression analysis enables a simple assessment of this unobserved network relative to the communication patterns of these actors. This helps us ask about a particular form of hierarchy which links campaign design with observed behaviors: are the most followed accounts also the most interacted with?

#### Quantifying communication strategies

Finally, we measure the engagement of account clusters in different communication strategies. In particular, we examine the relative proportion of tweets sent out by account clusters that constitute mentions, replies, and retweets.

To enhance comparability and distinctiveness between clusters, we run two normalization procedures. First, we convert raw counts of total mentions, replies, and retweets produced by a given cluster into proportions that sum to 1. Hence, *within* a given cluster that produced 1000 mentions, replies, and retweets each, we would first change these raw counts into 33%, 33%, and 33%, respectively. This allows us to fairly compare cluster behaviors since they may have widely varying total numbers of tweets.

Next, for each interaction type, we linearly scale these proportion scores *between* clusters, such that the cluster with the highest proportion of retweets is assigned a score of 100%, and the cluster with the lowest proportion of retweets is assigned a score of 0%. This maximizes distinctiveness, so that we can discern which clusters are more predisposed toward certain strategies over others.

All these procedures are formalized in Equation (5) below:

5 $$ s_{c,i} = \frac{\frac{r_{c,i}}{\sum_{j} r_{c,j}} - \min_{c'} \frac{r_{c',i}}{\sum_{j} r_{c',j}}}{\max_{c'}\frac{r_{c',i}}{\sum_{j} r_{c',j}} - \min_{c'} \frac{r_{c',i}}{\sum_{j} r_{c',j}}}, $$

where $r_{c,i}$ refers to the raw total number of times accounts in cluster *c* engage in behavior *i*∈ {mentions, replies, retweets}, and $s_{c,i}$ denotes the desired normalized value scaled from 0 to 100%.

## Results

Application of our integrated MVMC-based methodology revealed an organized system of state-sponsored accounts working together in groups engaged in distinct narrative and network maneuvers. In the following sections, we unpack their individual lines of effort and collective interrelationships. In sum, our analysis reveals that Chinese state-sponsored actors bore an overarching goal of managing Chinese international reputation. Over time, tactics deployed to achieve this underlying objective apparently became more diverse and sophisticated, specifically by augmenting (a) *destructive attacks against critics* with (b) *constructive projections of solidarity during the pandemic*.

Table 4 synthesizes the overarching themes in narrative and network maneuvers enacted by Chinese state-sponsored accounts. Following the literature on social cyber-security [2, 13], we classify the major information maneuvers according to previously well-studied strategic categories of: *boosting* influencers, *building* communities, producing *dismaying* negative messages, producing *enhancing* positive messages, and *distracting* publics with irrelevant or misleading content.

**Table 4:** Synthesis of MVMC-based mapping of Chinese state-sponsored account clusters

Strategic Theme	Narrative and Network Information Maneuvers	
	Time 1	Time 2
Hong Kong and Anti-Protest	- *Dismay*: Negatively frame protestors as violent and chaotic mobs - *Build and Enhance*: Expand group consensus around positive views of nation and police as sources of order - *Boost and Distract*: Masquerade as organic k-pop or health community	- *Dismay*: Negatively frame protestors and demand punishment - *Enhance*: Positively ask for peace to quell electoral chaos - *Boost and Distract*: Masquerade as organic community of Western music
Guo Wengui and Anti-Fugitive	- *Boost and Dismay*: Amplify organized community denouncing Guo as a liar and unreliable right-wing conspiracist	- *Dismay*: Denounce Guo as con man and self-interested fugitive
Pandemic Care and Community	(not yet present)	- *Boost and Enhance*: Amplify organized community of national strength and solidarity in time of crisis for China and Hong Kong

In the following sections, we unpack these overall results in three stages. First, we take a broad view of the set of state-sponsored accounts and map out their network of inter-cluster interaction to see their overall organization. Next, we drill down into each cluster’s use of hashtags and language to link psycholinguistic idiosyncrasies with particular narrative maneuvers. Finally, we measure the clusters’ levels of coordination, hierarchy, and relative preference for different communication methods to understand the varied ways they sought to influence networked information flow. Synthesizing these findings through the lens of social cyber-security, we show how distinct groups of accounts worked in unique yet complementary ways toward advancing the strategic goals of their overarching information operation.

### Overall cluster organization of Chinese state-sponsored accounts

**Figure 4:**
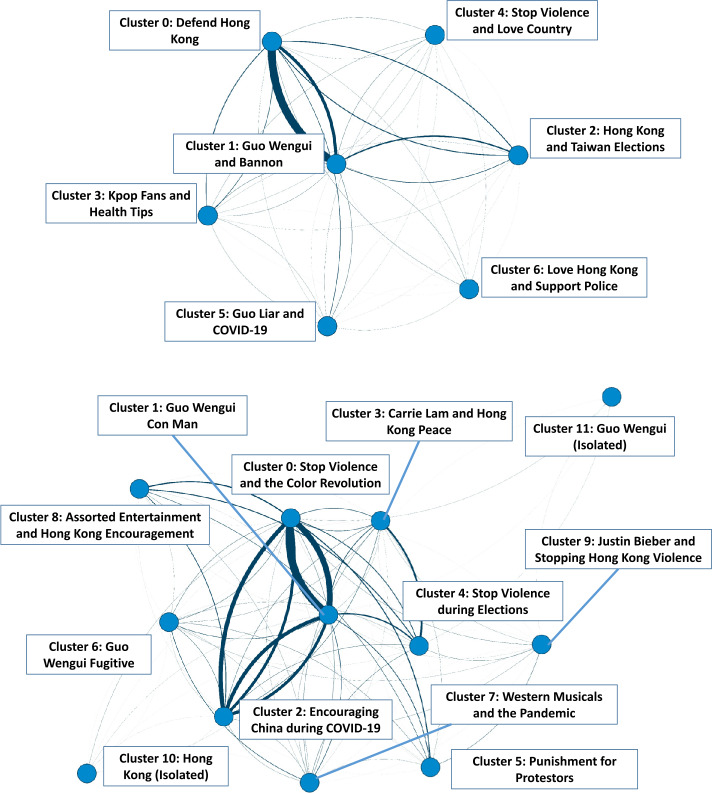
Organization of clusters of Chinese state-sponsored accounts, visualized on ORA. Nodes represent MVMC-derived clusters, connected by edges with thickness proportional to their weight. *Top:* Clustering obtained on accounts in Era 1. *Bottom:* Clustering obtained on accounts in Era 2

Figure 4 depicts the ORA visualization of the network of MVMC clusters derived for Eras 1 and 2. Descriptive statistics for each cluster are also provided in Table 5. MVMC results in Era 1 uncovered seven clusters with varied focus on Hong Kong and Taiwan, the fugitive billionaire Guo Wengui, and initial discourse on COVID-19. All clusters interacted with each other on some level, though majority of the inter-cluster interactions took place between Cluster 0 (Cluster Total Centrality = 0.094; Total Centrality Rank = 1), which focused on the Hong Kong protests and the message to “Defend Hong Kong”;^6^ and Cluster 1 (Cluster Total Centrality = 0.091, Total Centrality Rank = 2), which revolved around the topic of Guo Wengui’s partnership with Steve Bannon.^7^

**Table 5:** Descriptive statistics of derived MVMC clusters. Centralities are calculated on the cluster by cluster networks per time period

Era	Cluster	Size	Centrality (Total)	Centrality (Betweenness)
1	Cluster 0: Defend Hong Kong	3397	0.094	0.000
	Cluster 1: Guo Wengui and Bannon	2889	0.091	0.000
	Cluster 2: Hong Kong and Taiwan Elections	659	0.016	0.153
	Cluster 3: Kpop Fans and Health Tips	462	0.039	0.167
	Cluster 4: Stop Violence and Love Country	367	0.005	0.000
	Cluster 5: Guo Liar and COVID-19	313	0.008	0.000
	Cluster 6: Love Hong Kong and Support the Police	170	0.003	0.236
2	Cluster 0: Stop Violence and the Color Revolution	5945	0.060	0.083
	Cluster 1: Guo Wengui Con Man	5534	0.057	0.276
	Cluster 2: Encouraging China during COVID	2746	0.031	0.032
	Cluster 3: Carrie Lam and Hong Kong Peace	534	0.008	0.000
	Cluster 4: Stop Violence during Elections	433	0.005	0.192
	Cluster 5: Punishment for Protestors	369	0.004	0.058
	Cluster 6: Guo Wengui Fugitive	338	0.004	0.208
	Cluster 7: Western Musicals and the Pandemic	331	0.014	0.052
	Cluster 8: Assorted Entertainment and Hong Kong Encouragement	314	0.007	0.038
	Cluster 9: Justin Bieber and Stopping Hong Kong Violence	83	0.001	0.244
	Cluster 10: Hong Kong (Isolated)	38	0.000	0.321
	Cluster 11: Guo Wengui (Isolated)	15	0.000	0.173

Other clusters with lower total centrality had higher betweenness centrality, including Cluster 2 which focused on Hong Kong and Taiwan elections (Cluster Betweenness Centrality: 0.153; Betweenness Centrality Rank = 3); Cluster 3, which discussed Korean pop music and health tips (Cluster Betweenness Centrality = 0.167; Betweenness Centrality Rank = 3); and Cluster 6, which spread the message to love Hong Kong by supporting the police (Cluster Betweenness Centrality = 0.236; Betweenness Centrality Rank = 1). This overall structure revealed a highly centralized campaign among Chinese state-sponsored actors, featuring a set of core agents and narratives supplemented by more peripheral account clusters which act more as bridges between these key actor groups.

By contrast, in Era 2, we see a less centralized but more expansive system of twelve clusters. While general preoccupations with attacking Hong Kong and Guo Wengui persisted, activity related to the COVID-19 pandemic intensified. The overall structure of account clusters in Era 2 suggests an expanded set of core messages propagated by interrelated accounts belonging to Cluster 0 (Total Cluster Centrality = 0.060, Total Centrality Rank = 1) with its injunction to “Stop Violence” in Hong Kong; Cluster 1 (Total Cluster Centrality = 0.057, Total Centrality Rank = 2), with its denouncement of Guo Wengui as a “Con Man”; and Cluster 2 (Total Cluster Centrality = 0.031, Total Centrality Rank = 3), with its positive encouragement of Chinese resilience during the COVID-19 pandemic.

A larger number of peripheral groups (Total Centrality Range = [0.000, 0.014]) also contributed new narrative lines that supplemented these three core messages. As before, seemingly innocuous clusters had acted as bridges between actor groups, such as Cluster 7 which talked about Western musicals (Cluster Betweenness Centrality = 0.052; Betweenness Centrality Rank = 9); and Cluster 9, which discussed Justin Bieber (Cluster Betweenness Centrality = 0.244; Betweenness Centrality Rank = 3).

**Figure 5:**
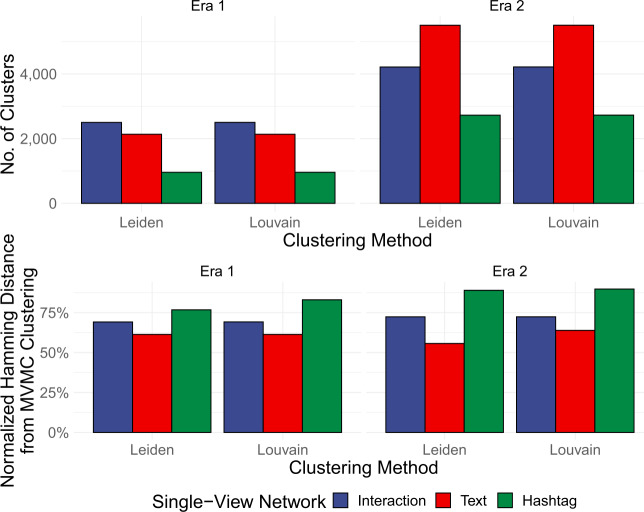
Comparison of MVMC clustering results with unimodal Louvain and Leiden baselines. *Top:* Baselines produce a significant number of degenerate clusters. *Bottom:* Baselines also capture only a fraction of coordinated accounts

As we shift to characterizing these clusters in greater detail, we note that the above partitioning of the state-sponsored actors using MVMC serves as a powerful and parsimonious advance over more traditional, unimodal methods. We compared results from state-of-the-art unimodal Leiden and Louvain clustering on single-view networks connected solely by account interactions, account text, and account hashtags [63, 64]. In general, as illustrated in Fig. 5 we saw that both Leiden and Louvain methods tended to produce orders of magnitude more degenerate clusters than MVMC did (mean: 3008.083; range: [959, 5507]), and clustered fewer accounts together than those which MVMC saw to be coordinated (mean: 71.99%; range: [55.68%, 89.77%]). Due to the single-view focus of prior methods, accounts that do not engage in every single form of coordination are not linked; MVMC addresses these issues through its multi-view approach.

### Characterizing narrative maneuvers

We now proceed to characterize these message systems in greater detail through the lens of narrative information maneuvers. We produced the above labels for each account cluster based on an analysis of their top-ranking and most distinctive hashtags, through which we obtained a qualitative sense of what messages each group seeks to propagate. Table 6 shows the list of hashtags associated with each account cluster based on both raw magnitude of use, as well as highlighting more distinctive hashtags based on the tf-idf score.

**Table 6:** Summary of hashtags used by MVMC clusters of Chinese state-sponsored accounts. Hashtags marked with a (*) are the hashtags with the top 3 highest tf-idf scores. Italics indicate hashtags did *not* undergo translation; otherwise they were in Chinese

Era	Cluster	Hashtags
Era 1	Cluster 0: Defend Hong Kong	Hong Kong, Mob, Defending Hong Kong, Protest*, Black Police, Defend Hong Kong, Hong Kong Police, Waste Youth*, Hong Kong Protests*, Guo Wengui
	Cluster 1: Guo Wengui and Bannon	Guo Wen Gui, Bannon, Hong Kong, Mob, Wang Yanping*, Defending Hong Kong, New York Times*, Wengui*, Protest, Hong Kong Police
	Cluster 2: Hong Kong and Taiwan Elections	Wang Liqiang*, Tsai Ingwen*, Hong Kong, Taiwan Election*, Hong Kong Election, Legislative Council, Guo Wengui, Hong Kong Human Rights And Democracy Act, Wang Liqiang, Defending Hong Kong
	Cluster 3: Kpop Fans and Health Tips	Hong Kong, Guo Wengui, Health Tips*, Hong Kong*, Twitter Best Fandom*, *Team BTS*, *Soompi Awards*, Charming China, Defending Hong Kong, HK
	Cluster 4: Stop Violence and Love Country	Hong Kong, Guo Wengui, Defend Hong Kong*, Stop Violence And Chaos*, Defend Hong Kong, Bannon, Mob, Police*, Hong Kong Police, Love The Country And Love Hong Kong
	Cluster 5: Guo Liar and COVID-19	Guo Wengui, Hong Kong, Pneumonia*, Fever*, Guo Liar*, *Miles Guo*, *Karaoke*, Fraud, *COVID19*, *Crypto*
	Cluster 6: Love Hong Kong and Support the Police	Guo Wengui*, Hong Kong, Guo*, Defending Hong Kong, Black Police, Love Hong Kong And Support The Police*, Hong Kong Election, Legislative Council, Bannon, Guo Wengui
Era 2	Cluster 0: Stop Violence and the Color Revolution	Hong Kong, Guowengui, Guarding Hong Kong, Police*, Pneumonia, Stop Violence And Control Chaos*, Virus, Mob, Color Revolution*, Color Revolution
	Cluster 1: Guo Wen Gui Con Man	Guo Wengui, Hong Kong, Miles Guo*, Bannon, Con Man*, Legal Fund*, Pneumonia, Wengui, Guo Liar, Pandemic
	Cluster 2: Encouraging China during COVID	Pneumonia, Hong Kong, Guo Wengui, Virus, COVID19*, Pandemic, Come On Wuhan*, Wuhan Epidemic*, Come On China, Novel Coronavirus
	Cluster 3: Carrie Lam and Hong Kong Peace	Hong Kong, Guo*, Old Monk Says Hong Kong*, Hong Kong*, Guo Wengui, Bannon, Carrie Lam, New York Times, I Say To Carrie Lam, Hong Kong Peace
	Cluster 4: Stop Violence during Elections	Hong Kong, Stop Violence And Control Chaos*, Defending Hong Kong*, Guo Wengui, Bannon*, Hong Kong Election, Legislative Council, Protests, Hong Kong Human Rights And Democracy Act, *HK*
	Cluster 5: Punishment for Protestors	Hong Kong, Mob, Protests*, Mobs Destroy Youth*, Severe Punishments For Mobs*, Cockroach, Defending Hong Kong, Hong Kong Protests, Hong Kong Mob, Defend Hong Kong
	Cluster 6: Guo Wengui Fugitive	Hong Kong, Hong Kong Election*, Legislative Council*, Hong Kong Human Rights and Democracy Act, Guo*, Guo Wengui, New York Times, Hypocrite, Wall Street, Fugitive
	Cluster 7: Western Musicals and the Pandemic	Guo Wengui*, Tootsie Musical*, Hong Kong, Guo Wengui, AMAs*, Guo Wengui, Pandemic, *The Prom Musical*, *Hong Kong*, *T Shirt Day*
	Cluster 8: Assorted Entertainment and Hong Kong Encouragement	Hala Madrid*, Hong Kong, Stop Violence And Control Chaos*, Love*, *Disney Plus*, Guo Wengui, Bannon, Come On Hong Kong, *Toyota*, *iSmart* *Shankar*
	Cluster 9: Justin Bieber and Stopping Hong Kong Violence	Hong Kong*, Hong Kong Garrison*, Stop Violence and Control Chaos*, Hong Kong Police, Port, *Janta Curfew*, *Evde Kal*, *Changes Out Now*, *Changes Tour*
	Cluster 10: Hong Kong (Isolated)	Hong Kong*
	Cluster 11: Guo Wengui (Isolated)	Guo Wengui*

For both Eras 1 and 2, we see that many hashtags are shared across virtually all clusters, including variants of Hong Kong and Guo Wengui. Hence, alongside the connectedness of the network in terms of inter-cluster communications, the consistency of messaging across account clusters also points to general narrative coordination across all accounts in the corpus. However, unique hashtags as well as hashtags with high tf-idf scores also reveal important nuances within shared narrative lines.

**Figure 6:**
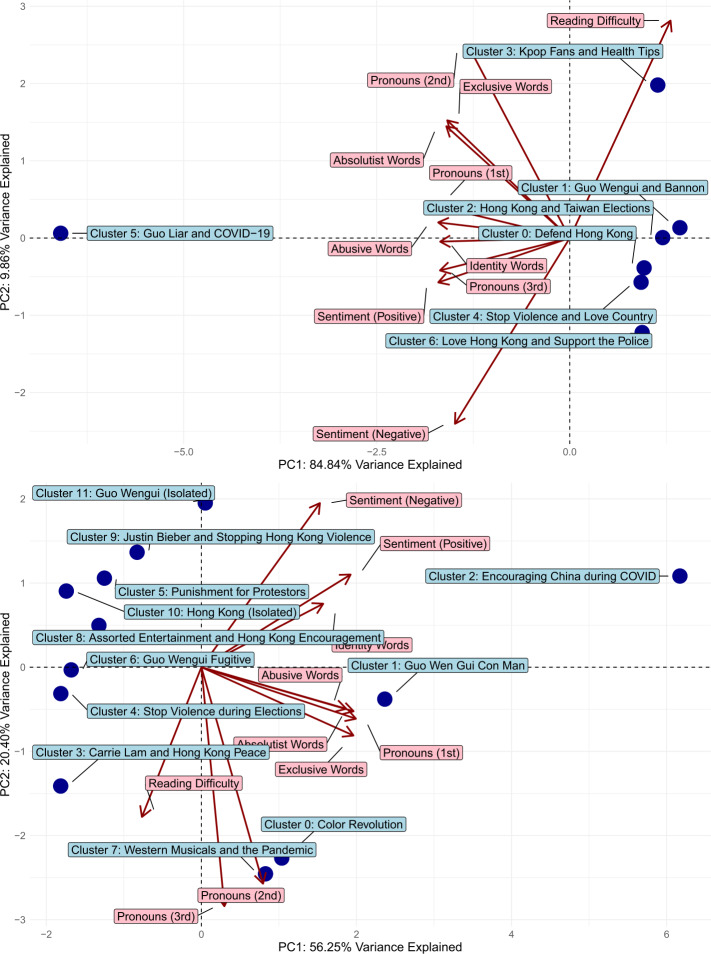
Two-dimensional visualization of variation in cluster-level psycholinguistic features based on PCA. Blue points depict the coordinates of account clusters based on the first two principal components. Red rays depict vector of each psycholinguistic feature based on the first two principal components. *Top:* PCA results in Era 1. *Bottom:* PCA results in Era 2

To push beyond a purely topic-based analysis, we also examine variations in psycholinguistic features across account clusters. This allows us to deepen our understanding of not just *what* account clusters discussed, but *how* they discussed them. Moreover, we map how account clusters’ messages related to each other in terms of shared and unshared psycholinguistic elements. Figure 6 shows the results of PCA over clusters and their associated psycholinguistic measures. Joint analysis of hashtags and psycholinguistic variation further sheds light on state-sponsored messaging around the following core themes.

#### Hong Kong and anti-protest narratives

In Era 1, we saw powerful, dismay-oriented narratives that painted Hong Kong protestors as “thugs” responsible for “violence and chaos” against which Hong Kong needed to be “defended” (Cluster 0; Cluster 4). Simultaneously, state-sponsored accounts sought to detract from support for the protestors by enjoining the public to “love” “the police” and “the country” (Cluster 4; Cluster 6). In this view, the core framing sought by the Chinese information operation aimed to contrast mass dissent in Hong Kong against a desirable ideal of peace and order. On the psycholinguistic map (Fig. 6), all three clusters were located in the bottom-right quadrant, closest to vectors of reading difficulty and negative sentiment – indicating that these narratives were communicated in terms of cognitively complex yet emotionally targeted attacks.

Interestingly, in Era 1, we also detected Cluster 3: an entertainment-oriented cluster that produced messages related to Korean pop music, Chinese television, and “health tips”, *alongside* the main anti-protest messaging of defending Hong Kong. While it is possible that these accounts may have simply been misclassified as state-sponsored accounts – as fandoms have been previously shown to be highly capable of coordinated activity [65] – psycholinguistic analysis in Fig. 6 suggests that this cluster actually produced the messages associated with the highest reading difficulty, while also producing Hong Kong and Guo Wengui messaging. So this may imply that popular culture is used here to masquerade as regular user activity while producing more cognitively complex messaging around key narrative lines of effort.

By Era 2, anti-protest frames became more sophisticated in their incorporation of additional narrative elements. Cluster 0 links the protests to the concept of the “color revolution”, a claim that historically associates anti-Chinese sentiment with alleged foreign influence, such as from the United States.^8^ Figure 6 shows strong psycholinguistic associations in this cluster with second-person pronouns as well as absolutist and exclusive words, which bolster the accusatory nature of this storyline. Aspirational narratives for peace and order additionally used ongoing elections in Hong Kong and Taiwan to bolster the need for social order (Cluster 3; Cluster 4). In this case, we saw strong association with reading difficulty, indicating that these storylines exhibited higher cognitive complexity in relation to their more intellectualized appeal for peaceful civic processes. Meanwhile, more explicitly negative attacks against protestors – featuring, as expected, a strong association with negative-sentiment terms – sought out severe “punishment” for them and dehumanizing them as “cockroaches” and faceless “mobs” (Cluster 5). Interestingly, a cluster which amplified Hong Kong anti-protest frames also used hashtags related to entertainment, specifically regarding Justin Bieber’s recent album release (Cluster 9). This cluster was also strongest in association with negative sentiment, suggesting that its use of popular culture may have again served a purpose of disguise or distraction, while sustaining anti-protest messaging.

#### Guo Wengui and anti-fugitive narratives

The second set of narratives which persisted throughout both eras had to do with the fugitive Chinese billionaire Guo Wengui. While we do not go into explicit detail about the complex history of Guo’s conflicts with the Chinese state, the narratives in Era 1 primarily sought to discredit him and his critical attacks against China.

Cluster 1 decried his associations with the prominent member of the American right-wing Steve Bannon, shortly after Bannon’s exit from the White House.^9^ Reading difficulty characterized this cluster as seen in Fig. 6, suggesting greater cognitive complexity in rejecting the legitimacy of Guo’s disclosures to the public about the inner workings of the Chinese government. Relatedly, in Cluster 2, state-sponsored actors also utilized cognitively complex rhetoric to undermine allegations by another self-confessed defector – Wang Liqiang – about Chinese attempts to influence Taiwanese and Hong Kong electoral politics. But in Cluster 5, we saw that state-sponsored actors harnessed less cognitively sophisticated messaging in discrediting Guo’s stories, especially when Guo’s allegations sought to link China to the initial rumors around the coronavirus. Messaging here shifted to become particularly rife with abusive and identity terms, which have been previously linked to online hate speech [7].

By Era 2, Guo-related narratives became less prominent and diverse, possibly because of a pivot to more pandemic-focused storylines which we subsequently explain. However, the same two major lines of narrative effort – cognitively explaining reasons for his untrustworthiness (Cluster 6) and hatefully denouncing him as a “liar” and a “con man” (Cluster 1) – nonetheless persisted.

#### Pandemic care and community narratives

Finally, we consider the novel emergence of narratives around care and community which became prominent in Era 2, around the time that the coronavirus was soon declared a global pandemic. In particular, Cluster 2 amplified messages encouraging Wuhan’s recovery from the virus, while Cluster 8 extended the same encouragement to Hong Kong. From a psycholinguistic standpoint, both clusters occupied the top-right quadrant of the PCA map in Fig. 6, and were strongly associated with both positive-sentiment and identity-related terms.

This suggests that another central facet of the Chinese state-sponsored information operation sought to protect its international reputation not only by discrediting individual (Guo Wengui) and collective (Hong Kong protestors) opponents. Projecting a positive sense of national solidarity was also vital to managing China’s influence on the international stage, especially amidst a global crisis.

### Characterizing network maneuvers

Having established several main threads of Chinese state-sponsored messaging, we now proceed to characterizing their associated network maneuvers. In this section, we ask: How hierarchically coordinated were account clusters? What communication strategies did they use to achieve their strategic goals?

**Figure 7:**
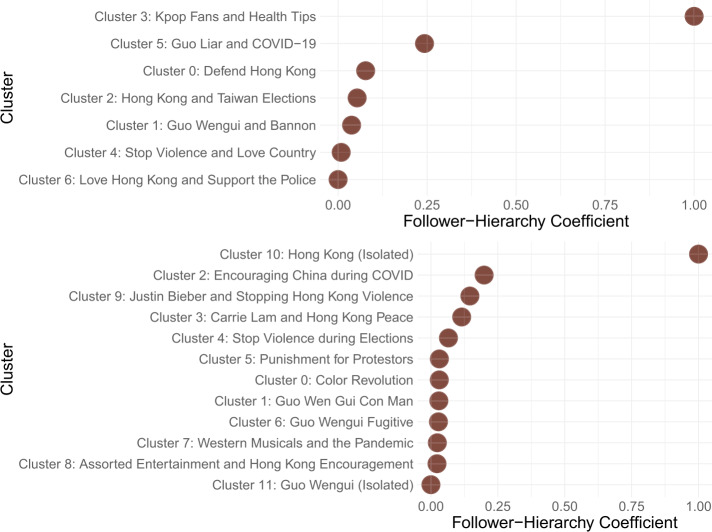
Follower-hierarchy coefficients for account clusters normalized to values between 0 and 1. Most clusters feature similar levels of hierarchical organization, with a few outliers exhibiting much stronger hierarchy. *Top:* Results for Era 1. *Bottom:* Results for Era 2

**Figure 8:**
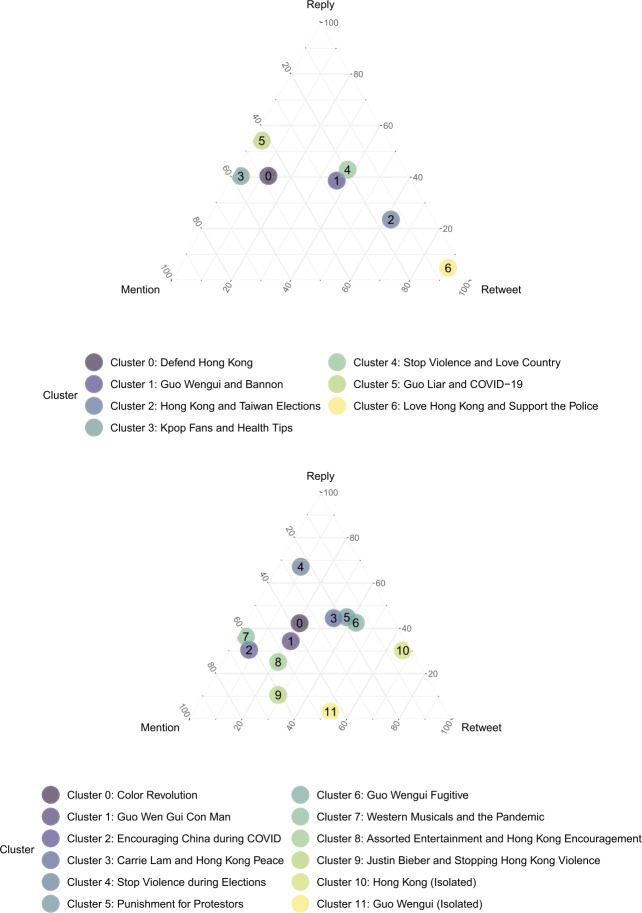
Relative interaction preferences for account clusters normalized to values between 0 and 1. Most clusters specialize in mentions and replies, with a few outliers specializing in retweets. *Top:* Results for Era 1. *Bottom:* Results for Era 2

Figure 7 offers insight into the first question. Here, we plot follower-hierarchy coefficients that quantify the extent to which the most-followed accounts in each cluster also controlled the most information flow as measured by total-degree centrality. Meanwhile, for the second question, we turn to Fig. 8, which shows the relative preferences of each cluster for mentions, retweets, and replies. Once again, joint analysis of hierarchical organization and communication strategies offer holistic insights into the network maneuvers undertaken by state-sponsored actors. As we unpack below, three broad behavioral themes were particularly striking among the derived account clusters.

#### Balanced operators

For both Eras 1 and 2, the core messages around Hong Kong and Guo Wengui featured moderate levels of hierarchical organization, especially Clusters 0 and 1 in Era 1, and Clusters 0 and 1 in Era 2. Behaviorally, both pairs of clusters were also located near the center of Fig. 8, denoting a relative balance in terms of retweets, replies, and mentions. This suggests that for these two core narrative lines of effort, their role in the overall information operation was consistent over time: to balance a combination of amplifying selected messages and interacting with other accounts. Moreover, these lines of effort did not appear to demonstrate strict leader-follower dynamics, although some level of hierarchy may have been observed due to varying levels of success in gaining influence.

#### Organized masqueraders

Very interestingly, some of the most hierarchically organized clusters in Era 1 were Cluster 3, which dealt with k-pop fans and health tips; and Cluster 5, which dealt with denouncing Guo Wengui as a liar. Meanwhile, in Era 2, some of the most hierarchically organized clusters were Cluster 2, which sought to encourage China during the pandemic, and Cluster 9, which discussed Hong Kong violence alongside pop culture references to Justin Bieber. While featuring similar network organization, these clusters also narratively shared messages that deviated from core attack storylines, including references to pop culture and entertainment. Behaviorally, these are also some of the accounts utilizing the highest rates of mentions and replies, with virtually no retweets. Taken together, this evidence seems to suggest that one of the important network tactics used in the information operation involved structuring information flow in a manner that enabled masquerading as human – that is, through more conscious interactions with others, and by mimicking more natural leader-follower dynamics, concentrating influence among actors most reasonable to possess it. The cluster projecting national solidarity during crisis, in particular, may have benefited the most from this seemingly organic set of actors.

#### Egalitarian echo-chambers

Interestingly, across eras, some of the least hierarchically coordinated clusters were those in Era 1 which pushed positive frames of loving the country and supporting the police to dampen sympathies for the Hong Kong protestors (Cluster 4 and 6). These clusters also used some of the most retweets relative to the rest of the dataset. This may indicate that, in Era 1, these messages were not yet delivered with particularly concerted leader-follower dynamics. Instead, they were organized primarily to amplify this narrative frame across a swath of accounts, tantamount to the creation of an echo-chamber effect. Such an egalitarian structure for information flow, in this view, would potentially be beneficial for such a narrative strategy, especially as its objective is not necessarily to communicate hierarchy, but simply the appearance of consensus to quash burgeoning international support for anti-Chinese civil dissent in Hong Kong.

#### Remarks on outliers

Finally, we remark on a few outliers. In Era 2, we noticed that the most hierarchically organized cluster was a relatively isolated set of 38 accounts that tweeted only using a single hashtag related to Hong Kong. Conversely, the least hierarchically organized cluster was also an isolated set of 15 accounts that tweeted only using a single hashtag related to Guo Wengui. These outlier examples are interesting in that they also relied primarily on retweets as opposed to interactions like mentions and replies. This may indicate that for some subsets of the information operation, their function may simply be to amplify content from other sources – whether in a concentrated, hierarchical fashion or in a relatively egalitarian fashion. They may also signal a fragmented set of unsuccessful accounts in the overall information operation that simply did not accrue enough meaningful influence despite their activities captured in the data.

## Conclusions

This paper introduced and applied a computational framework for mapping the strategic organization of state-sponsored information operations. Based on an unsupervised multi-view modularity clustering technique, we identified core and peripheral state-sponsored accounts seeking to manage Chinese reputation on the international stage.

Collectively, our findings point to a coordinated system of state-sponsored accounts advancing interrelated state interests. State-sponsored accounts work toward these objectives through diverse yet complementary tactics that take advantage of social media’s large-scale messaging and interaction features. More specifically, the case we examine illustrates how a single information operation may employ *both* positive and negative messaging (i.e., to implement a carrot-and-stick strategy), as well as *both* hierarchical and egalitarian strategies (i.e., to communicate leadership versus consensus) in order to achieve different aspects of an overarching set of campaign objectives. This work thus pushes existing computational efforts to go beyond simple binaries in information operations research. It is crucial to inquire into not just who is or is not manipulative (e.g., bots or trolls), but also *what kinds* of manipulation may take place, and how these multiple lines of effort coalesce within a wider digital campaign.

This sets our work apart from the rich work in this area, which often seeks to use supervised machine learning for information operation detection [8, 66, 67]. The commonality that cuts across much of this scholarship is their analysis of inorganic accounts as relatively homogeneous in nature, eliding the differential functional units influence campaigns may be divided into in conducting particular information maneuvers. We affirm that there is certainly landmark value in distinguishing accounts primarily at the level of organic versus inorganic. Work that begins from this framework valuably exposes general patterns of activity among inorganic accounts [29, 38, 54], demonstrates different means of measuring such coordination [9, 19, 20], and shows the extent and limits of inorganic influence on online conversations [35, 42, 68]. What our approach contributes is a method for considering holistically how an information operation is designed and orchestrated by performing *heterogeneous* functions, and identifying how those functions interrelate.

From a methodological standpoint, our approach thus contributes to applications of unsupervised methods for understanding information operations in particular, as well as coordinated activity in general [9, 19, 20]. We advance the social cyber-security use of clustering by adopting an intermediate integration and multi-view framework [16, 21]. Moreover, we highlight the significance of mapping coordination *within* a set of state-sponsored accounts, rather than looking to distinguish *between* organic and inorganic activity. This further highlights the advances of our work relative to related scholarship that relies mainly on more traditional, unimodal clustering techniques; or uses clustering primarily to separate inorganic accounts from organic ones, without probing the diversity that may be salient among a set of accounts belonging to a given information operation.

In addition, we develop principled tools for probing the derived clusters to extract their role in the wider “group of groups” that make up an information operation. This latter contribution is vital, as it aids in the qualitative assessment of strategic organization, by lending more precise quantitative evidence for analysts to take advantage of. Moreover, we point out that the advances presented by MVMC here do not lie in opposition to existing tools, but may in fact readily incorporate their signals as new views in the clustering process. For instance, richer analyses of information operations could potentially be facilitated by further adding predictions from supervised models as network views, including scores for automation [8, 11], hate speech [7, 54], or fake news [10].

Beyond methodological innovations, this paper also suggests substantive insights (and questions) for scholarship at the intersections of international politics, social media, and platform regulation. In particular, it speaks to the complicated and increasingly salient role played by online platforms for not only facilitating large-scale public perception and meaning-making, but also reshaping them – with potentially harmful consequences [5, 26].

In view of our multi-directional findings on Chinese narrative and network maneuvers, our call to shift beyond binary definitions of malicious content or activity also extends to practice and policy. We posit the need to confront more nuanced questions about the broader political contours that may attend seemingly benign – even overtly positive – messaging and behaviors [23]. Additional computational and policy scholarship may also be vital to understand the role played by online communities that are not explicitly designed for political communication or organization, yet may nonetheless be instrumentalized for such ends [65]. Such publics (e.g., pop culture, health groups) are ubiquitous, diverse, and legitimate participants in online discourse. How can platforms be designed to prevent their manipulation, without curtailing their free and open participation in digital spaces? And how can platforms be accountable, or enforce accountability to concerted state-backed efforts to shift public opinion – even for arguably rehabilitative rather than regressive purposes? Our work opens up partial answers to these queries, yet further work remains to be done.

### Limitations and future work

In looking ahead to future work, we remark on a number of key limitations of the present approach. First, our analysis was naturally constrained by the availability of data as shared in Twitter’s information operation disclosure framework. It is possible that incorporating other sources of information – whether within the social media platform, or other external signals – may shed further light on the activity and objectives of information operations. That said, while we affirm the richness of our analysis based primarily on text and interaction views, we also note that Twitter disclosures also provided highly informative data on URL-sharing and multimedia messages [42]. Future work may tap into these features as additional views to inform even more nuanced analysis of narrative and network maneuvers.

Speaking to the flexibility and generality of our methods [12], our overarching framework can further be readily adapted and modified to apply to other information operations (e.g., by other state actors), as well as coordinated campaigns in general. In particular, future research may aim to apply this methodological framework to any number of the datasets disclosed by Twitter, as the methods were specifically designed to work in a generalizable manner across different languages and societal settings. Investigative work seeking more in-depth analysis of a particular campaign, however, may augment our approach by exploring more advanced methods at any juncture of our analysis, such as higher-level natural language processing techniques to drill down on narrative maneuvers, or graph mining tools to further characterize network maneuvers.

Another limitation in this work lies in its use of a retrospective dataset of already suspended accounts. Here, our more forensically oriented analysis does not directly implement a real-time framework for detecting and characterizing ongoing information operations [8, 28]. A well-motivated extension of our approach would then integrate existing efforts in the literature to use clustering for separating human and inorganic accounts, while drawing insights from our work to map out the latter’s coordinated organization. Relatedly, it would also be intriguing to consider the differences in how multi-view network topologies differ between inorganic and organic accounts.

Finally, while we empirically characterized narrative and network maneuvers, which may speak to strategic *intent*, a secondary analysis of human *impacts* of these strategies was beyond the scope of this study [35]. As noted by Twitter,^10^ the accounts in this dataset were “largely caught early and failed to achieve considerable traction on the service”. This helps us narrow our analytical insights into how these inorganic accounts were designed in relation to each other in forwarding narrative and network maneuvers, as by the platform’s own telling, limited organic reach was obtained by these operations. Hence, while our work may shed light on the intended campaign design of state-sponsored operations, they do not speak directly to the type of influence such campaigns successfully achieve. Other research methods – including surveys, experiments, or even ethnographic work – could provide complementary insight into how believable or effective the narrative and network strategies we observed may have been.

## Data Availability

Data is made publicly available by Twitter at https://blog.twitter.com/en_us/topics/company/2020/information-operations-june-2020.html. Code to run the MVMC algorithm is also publicly available at https://github.com/ijcruic/Multi-view-Clustering-of-Social-Based-Data.

## Footnotes

1 https://blog.twitter.com/en_us/topics/company/2020/information-operations-june-2020.html

2 https://www.nytimes.com/2019/11/02/world/asia/hong-kong-protests.html?searchResultPosition=13

3 https://netanomics.com/ora-pro/

4 https://netanomics.com/netmapper/

5 Code is available at https://github.com/ijcruic/Multi-view-Clustering-of-Social-Based-Data

6 https://www.nytimes.com/2020/05/23/world/asia/china-hong-kong-propaganda.html

7 https://www.nytimes.com/2019/12/23/us/politics/steve-bannon-guo-wengui.html

8 https://thediplomat.com/2021/05/hong-kongs-new-police-state/

9 https://www.nytimes.com/2020/11/20/business/media/steve-bannon-china.html

10 https://blog.twitter.com/en_us/topics/company/2020/information-operations-june-2020

## Abbreviations

COVID-19: Coronavirus Disease 2019
MVMC: Multi-View Modularity Clustering
ORA: Organization Risk Analyzer
PCA: Principal Component Analysis
tf-idf: Term Frequency Inverse Document Frequency
